# Enlargement of the Prostate.—I

**Published:** 1909-05-08

**Authors:** 


### SURGERY.

### ENLARGEMENT OF THE PROSTATE.—I.

ENLARGEMENT of the prostate is an affection which is confined to the later decades of life. It is rarely met with in men under the age of fifty. In recent years, since the operation of prostatectomy has been more extensively practised, and since therefore one has been able to examine the exact condition in a great number of cases, it has come to be recognised that a large variety of different conditions are responsible for the symptoms which are classed under the title "enlargement of the prostate."

In fact, a genuine hypertrophy of the gland, in which all the elements composing its structure are included, is the exception rather than the rule—*i.e.* an enlargement that can be regarded as analogous to a parenchymatous goitre. The enlargement is more often in the nature of a fibrosis, and the most reasonable hypothesis which can be put forward to account for this condition is that it is the result of a chronic prostatitis, possibly from a gonococcal infection many years previously. In these cases the prostate may be little, if at all, larger than normal, owing to the contraction of the fibrous tissue; yet all the symptoms of enlargement of the prostate will be present owing to a distortion of the prostatic urethra. From an operative point of view, the enucleation of such a prostate presents more than ordinary difficulty.

In other cases the enlargement is due to the formation of a benign tumour in the gland, and both adenoma and fibromyoma have been described. Since the prostate is a sexual organ, and is the analogue in the male of the uterus in the female, one might expect a fibromyoma to be a common occurrence; and yet experience shows that this is not the case. Fibromyoma of the prostate is comparatively rare. A simple adenoma, when it occurs, generally causes a localised enlargement of one lateral lobe, thus distorting the urethra and giving rise to the symptoms about to be described. It is only when the adenoma is localised and encapsuled that the obstructing mass can be removed without interfering with the continuity of the prostatic urethra. Carcinoma is also a cause of enlargement of the prostate, and when it occurs starts in the glandular tissue of the organ. Yet this is not always so. In a case recently seen by the writer, the patient, a man of seventy-one, was undoubtedly suffering from symptoms referable to enlargement of the prostate. Rectal examination revealed a hard mass pressing back on the anterior wall of the bowel, and the bladder contained four ounces of residual urine. The bladder was opened by suprapubic cystotomy, and the prostate could be felt forming a prominent projection into the base of the bladder. It was enucleated. The gland itself was apparently normal, but it lay perched upon a bed of hard tissue. Some of this was also removed. Microscopical examination showed that the prostate itself was normal, but that there was malignant disease of the peri-prostatic capsule.

There remains yet another variety of enlarged prostate, the so-called "enlarged middle lobe," on which great stress was laid in the days when our knowledge of this condition was less perfect. The real state of affairs found in the cases described under this heading is usually a polypoid excrescence growing from one of the lateral lobes, either a pedunculated adenoma or an outgrowth of granulation tissue, which falls over and occludes the orifice of the urethra by a species of ball-valve action, in much the same way as a pedunculated growth from the pelvis of the kidney may occlude the opening of the ureter. In these cases the symptoms are somewhat different, the patient complaining of periodical attacks of retention of urine, requiring the passage of a catheter.

The pathological changes are easily understood. The first result of enlargement of the prostate is obstruction to the outflow of urine; and hypertrophy of the bladder, as shown by fasciculation of its walls. But soon even the hypertrophied bladder is unable to do its work, and hypertrophy is succeeded by dilatation, as happens in other hollow viscera whose outlet is obstructed. The same phenomena are seen in stenosis of the pylorus. In cases which have been left unrelieved for any length of time, the ureters may also become dilated and hydronephrosis may supervene. This, however, is not common. The enlarged prostate forms a projection in the base of the bladder, and behind this a cavity is found in which urine is retained after the bladder has apparently been emptied by natural means. The urine so retained is known as "residual urine."

The first thing which the patient notices is increased frequency of micturition, and he is disturbed at night by the necessity of getting up to pass his water. This is followed by increased urgency of micturition; he feels that as soon as the desire to micturate comes upon him, he is unable to delay; and yet when he does try to micturate, the flow does not come at the beginning of the attempt. When it does come the power of projection is diminished, the act takes unduly long, and there is some dribbling at the end. Even then he is left with the sensation that he has incompletely emptied his bladder.

As the disease advances all these symptoms increase in severity, and there comes a time at last when his urine is continually dribbling away from him, and his life is made unpleasant by the constant presence on his clothes of urine which is undergoing ammoniacal degeneration.

In many cases there is occasional hæmaturia, even though no catheter has been passed. This is due to the fact that the enlarged prostate is engorged and bleeds readily, and the contractions of the bladder at the end of micturition are sometimes sufficient to produce this effect of their own accord.

Small clots may thus be passed, and have been known to become impacted in the urethra, generally at the narrowest point opposite the corona glandis, leading to absolute retention of urine. If recognised, the condition is easy to treat. Firm but steady pressure with a gum elastic catheter will dislodge them.

